# Case Report: Cytologic Description of Somatotroph Pituitary Adenoma in a Cat

**DOI:** 10.3389/fvets.2022.934009

**Published:** 2022-07-18

**Authors:** Flavio H. Alonso, Kevin D. Niedringhaus, Mariah G. Ceregatti, Marisa A. Maglaty

**Affiliations:** ^1^Veterinary Medical Teaching Hospital, School of Veterinary Medicine, University of California, Davis, Davis, CA, United States; ^2^Department of Biomedical Sciences, Ross University School of Veterinary Medicine, Basseterre, Saint Kitts and Nevis; ^3^Department of Veterinary Clinic and Surgery, School of Veterinary Medicine, Federal University of Minas Gerais, Belo Horizonte, Brazil

**Keywords:** brain, histopathology, central hypothyroidism, hypophysis, diabetes mellitus

## Abstract

This case report describes for the first time the cytologic characteristics of a hormonally secreting pituitary adenoma in a cat. An 8-year-old female spayed domestic long-haired cat was referred with a previous diagnosis of hypersomatotropism and secondary diabetes mellitus 7 months prior. Clinical signs included weight loss, polyphagia, polyuria, and polydipsia. Serum insulin-like growth factor-1 was 340 nmol/L (RI: 12-92), and CT scan revealed a hypophyseal mass, and a presumptive diagnosis of acromegaly was made. A transsphenoidal hypophysectomy was performed. A fragment of the pituitary gland was subjected to a squash preparation and cytology revealed a neuroendocrine neoplasm characterized by anisokaryosis and prominent nucleoli. Additional cytologic findings included cell cohesiveness, indistinct cytoplasmic borders, nuclear crowding, molding, and fragmentation. A diagnosis of adenoma was based on a lack of histopathologic or imaging evidence of invasion. A week later, during post-surgical hospitalization, the patient worsened and died. Histopathology from a necropsy procedure revealed fibrinosuppurative meningitis as a post-surgical complication. Pituitary adenomas might have an aggressive cytologic appearance, despite a lack of histopathologic invasion or dissemination.

## Introduction

The prevalence of pituitary adenomas varies from 15 to 20% in people (1, 2) and represented 30 to 61% of the pituitary masses found in dogs in multiple studies (3–5). These can be classified based on size (microadenomas when they are smaller than 10 mm and macroadenomas when 10 mm or larger) and secretory pattern (e.g., corticotroph, somatotroph, melanotroph, or plurihormonal) (6). Somatotroph adenomas represent the most common pituitary tumor in cats and, when associated with hypersomatotropism, present with weight gain, broad facial and limb features, abdominal organomegaly, heart murmur, and diabetes mellitus-related clinical signs (7–9). In a recent study (9), the median survival time in cats with pituitary adenomas varied from 515 to 730 days, depending on the tumor type (9). Diagnosis and treatment normally depend on clinical signs, advanced imaging investigation, histopathology, transsphenoidal surgery, medical management, or radiotherapy (6, 10).

Anatomic and histopathologic findings have been extensively described in animals, but scientific reports approaching the cytologic features of pituitary neoplasms are still lacking. The objective of this report is to describe the findings of an intraoperative imprint smear cytology from a somatotroph pituitary adenoma in a cat, in the context of a complete diagnostic work-up protocol. This case report was written in agreement with the most updated version of the CARE guidelines (11).

## Case Description

An 8-year-old female spayed domestic long-haired cat was referred to the neurology and neurosurgery service of the UC Davis William Pritchard Veterinary Medical Teaching Hospital for transsphenoidal hypophysectomy. The patient received a diagnosis of hypersomatotropism (serum insulin-like growth factor-1 = 340 nmol/L, RI = 12–92) and secondary diabetes mellitus 7 months prior to admission. Insulin therapy had been discontinued for about 6 months, and since then, the patient had lost ~680 g in weight and developed polyuria and polydipsia. On physical examination, the patient weighed 3 kg (body condition score = 3/9), and was bright, alert, responsive, and hydrated. Prognathism and prominent facial features were noted. No apparent cardiovascular, respiratory, integumentary, gastrointestinal, or neurologic abnormalities were noted, and no relevant familial history was reported.

Prior to the surgical procedure, a complete blood count (CBC, Advia 120 hematology analyzer; Siemens) revealed moderate lymphopenia (486 lymphocytes/μl, RI = 1,000–7,000), and slight thrombocytosis (576,000 platelets/μl, RI = 180,000–500,000). The biochemical panel (Cobas 6000 C501; Roche) revealed mixed metabolic acidosis and alkalosis (anion gap = 31 mmol/L, RI = 13–27 and bicarbonate = 19 mmol/L, RI = 15–21), selective chloride loss (chloride = 101 mmol/L, RI = 117–126 and sodium = 145 mmol/L, RI = 151–158), mild hyperkalemia (5.5 mmol/L, RI = 3.6–4.9), marked hyperglycemia (560 mg/dL, RI = 63–118) and mild hypertriglyceridemia (129 mg/dL, RI = 8–80). Serum fructosamine levels were 683 μmol/L (RI = 190–365) and a thyroid panel (Immulite 2000; Siemens) revealed total T4 < 0.5 μg/dL (RI = 1.1–3.3), free T4 <0.3 ng/dL (RI = 0.59–3.04) and TSH <0.03 (RI = 0-0.07). Urinalysis was performed on a sample obtained by cystocentesis, which revealed marked glucosuria, mild proteinuria and hypersthenuria (USG = 1.036, RI > 1.035).

**Figure 1:**
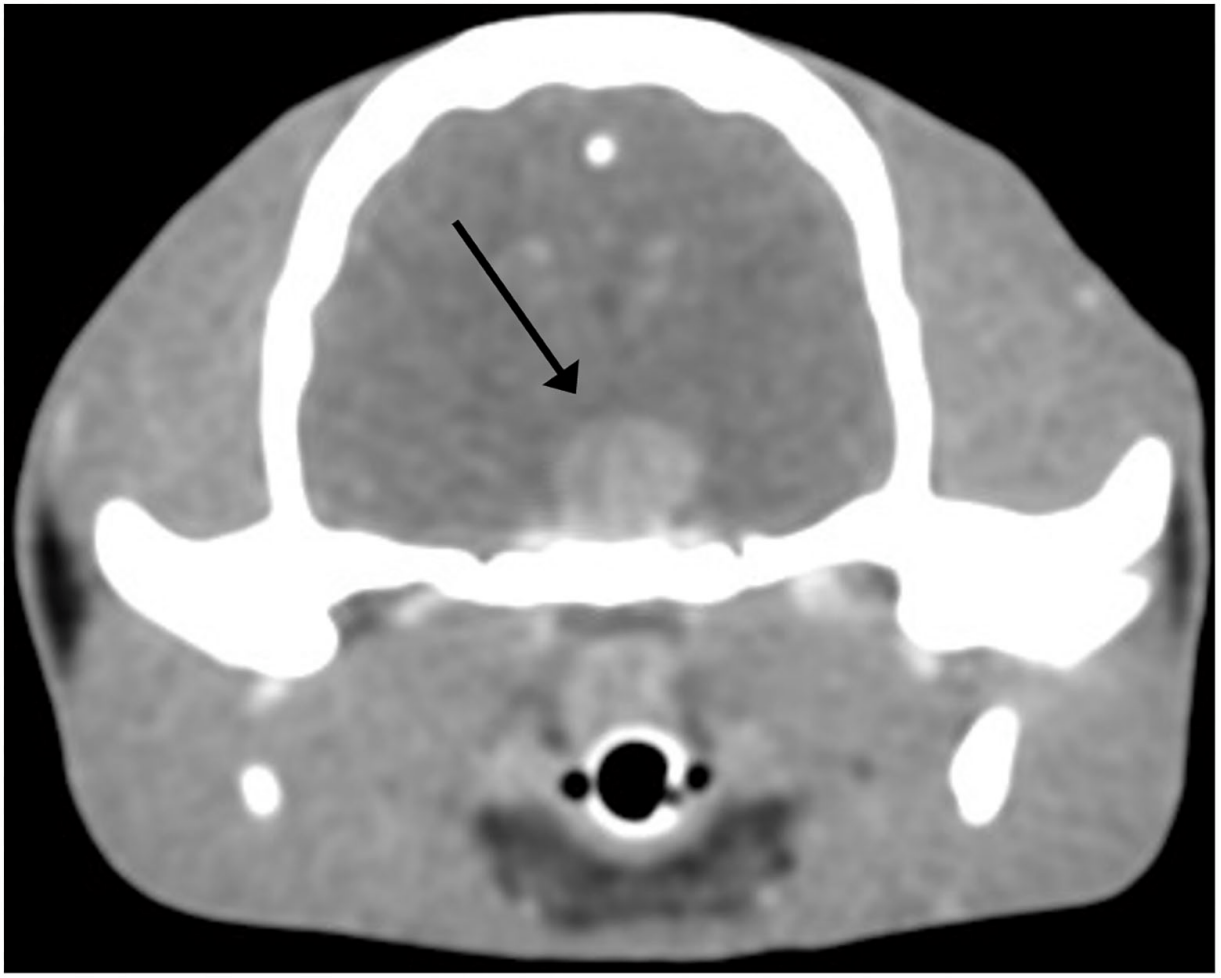
Pituitary adenoma, cat. Postcontrast transverse CT image of the skull in soft tissue window. An 8.6 by 8.3 by 6.8 mm mass (arrow) is noted at the anatomical region of the pituitary gland. A slice thickness of 0.62 mm was used.

Pre-surgical transverse contrast-enhanced CT images of the brain revealed a hyperintense large ovoid-shaped mass in the area of the pituitary gland measuring 8.6 by 8.3 by 6.8 mm (Figure 1).

**Figure 2:**
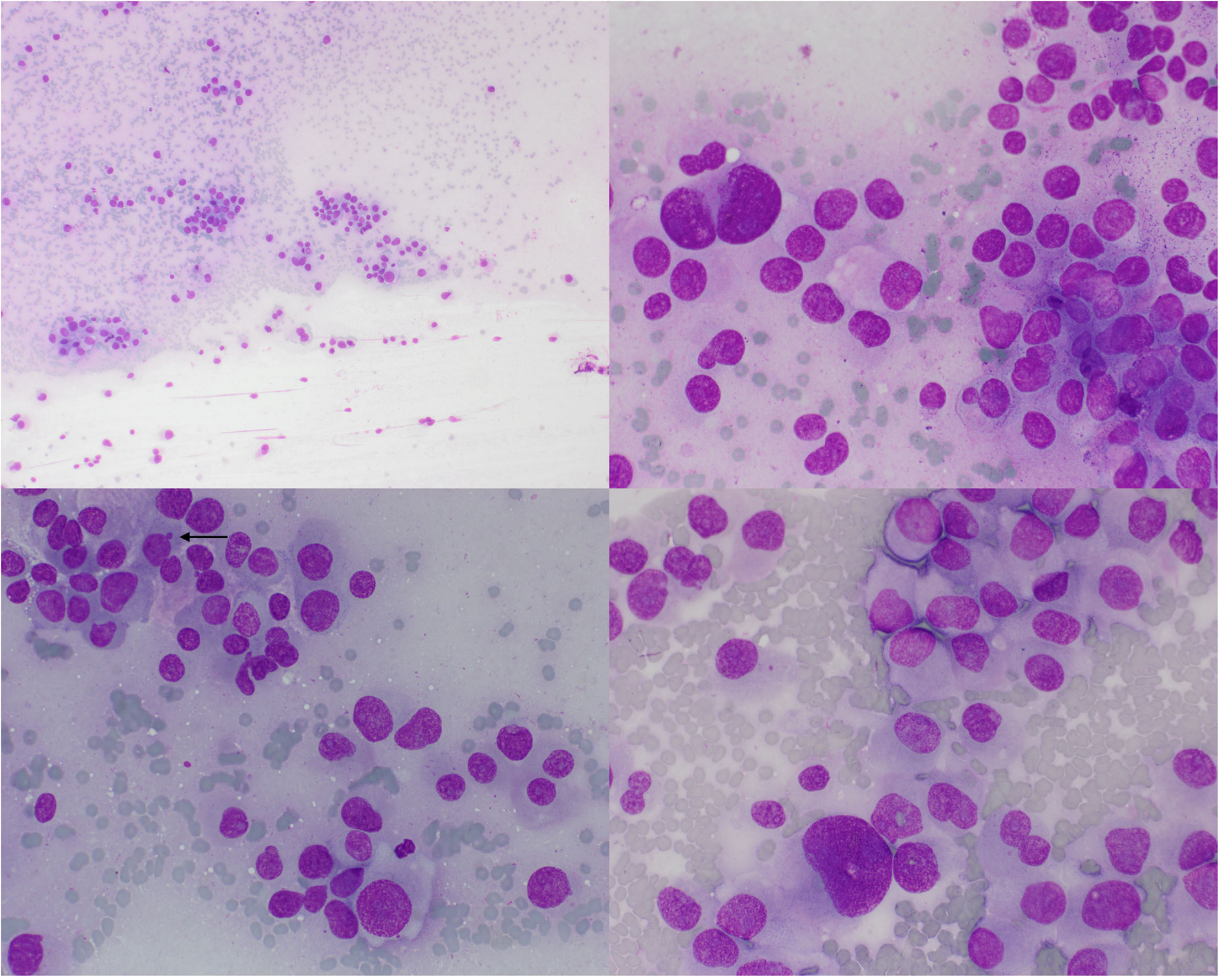
Pituitary adenoma, cat. An imprint smear of the surgically excised pituitary mass revealed moderate numbers of neuroendocrine neoplastic cells arranged individually or in small aggregates. Cells have round to pleomorphic nuclei and a pale gray cytoplasm with poorly distinct borders and occasional vacuolization. The chromatin is finely stippled and contains multiple prominent nucleoli. Anisokaryosis is moderate to marked and occasional micronuclei are noted (arrow). Wright's-Giemsa, 10 × (upper left) and 50 × objective lenses.

During the transsphenoidal hypophysectomy, imprint smears from the pituitary mass were prepared and submitted for cytologic analysis. The specimen was highly cellular, and the background contained a diffuse and amorphous light pink material (consistent with fragmented cytoplasms) and a low number of red blood cells, admixed with many naked nuclei and other cellular debris. Intact nucleated cells were scattered throughout in vaguely cohesive sheets and clusters and presented indistinct borders, intermediate nucleus:cytoplasm (N:C) ratio, and a pale blue to pink smooth cytoplasm. The nuclei of this population of cells were oval to polygonal with finely granular chromatin patterns and multiple small and prominent nucleoli. Anisokaryosis and anisocytosis were marked. Nuclear crowding, molding, and fragmentation were noted. Rare endothelial cells were also noted. Findings were interpreted as neuroendocrine neoplasia (Figure 2).

**Figure 3:**
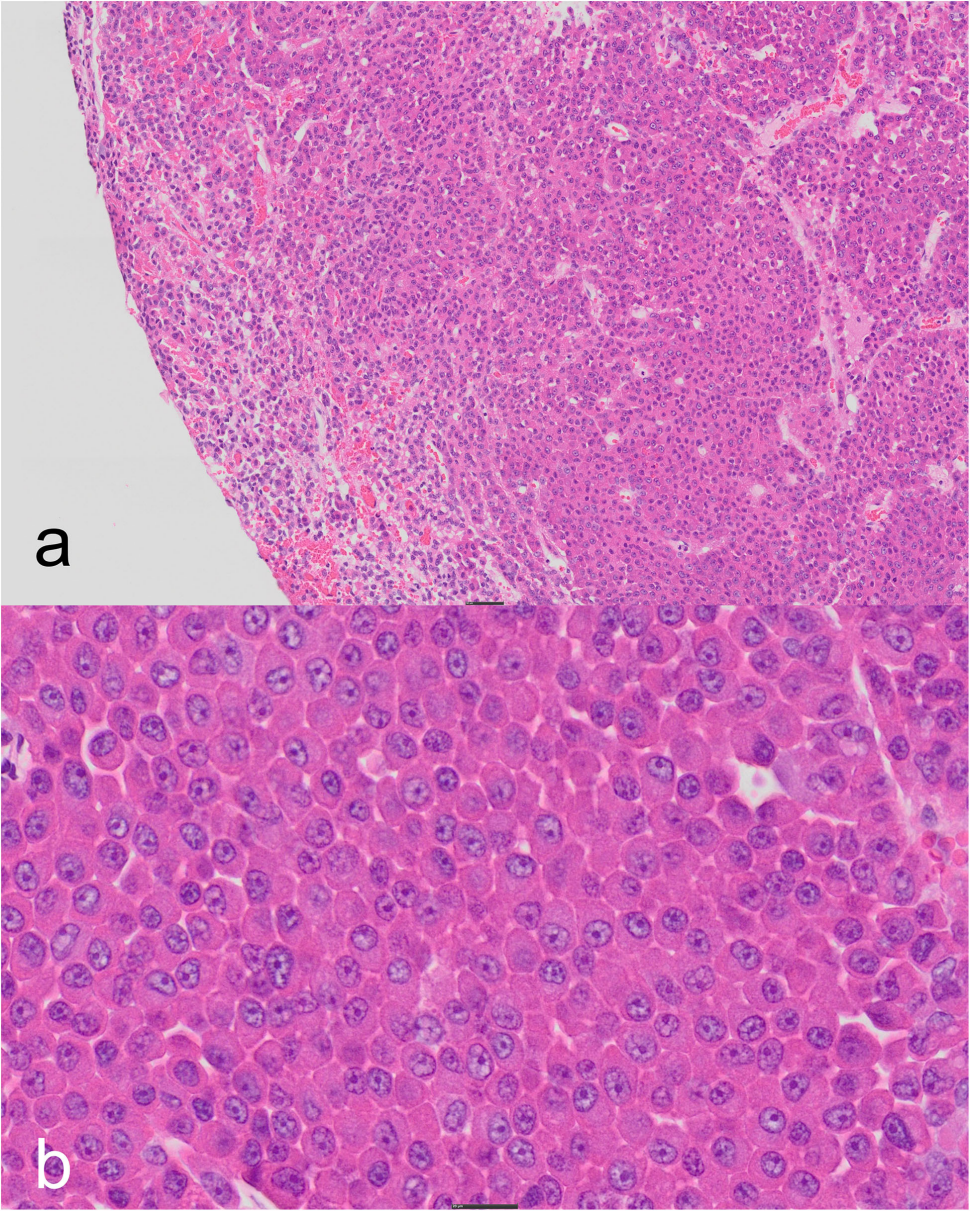
Pituitary adenoma, cat. **(a)** The center of the tissue contains a non-encapsulated mass consisting of nests and cords of a monomorphic population of cells compressing the peripheral parenchyma with scant, acute hemorrhage (4 × objective lens). **(b)** Densely-packed sheets of well-differentiated, brightly eosinophilic, granular cells with large central nuclei and prominent, single nucleoli (20 × objective lens). Hematoxylin-eosin.

On histopathologic evaluation, the pituitary gland tissue consisted of a densely cellular, expansile, unencapsulated mass composed of nests and cords of polygonal cells on a fine fibrovascular stroma. Neoplastic cells presented distinct cell borders and a moderate amount of brightly eosinophilic glassy to granular cytoplasm. Nuclei were round with vesiculated chromatin and one prominent nucleolus. Anisocytosis and anisokaryosis were mild. Mitotic figures were rare (Figure 3). The periphery revealed compression and mild atrophy of adjacent, non-neoplastic tissue.

**Figure 4:**
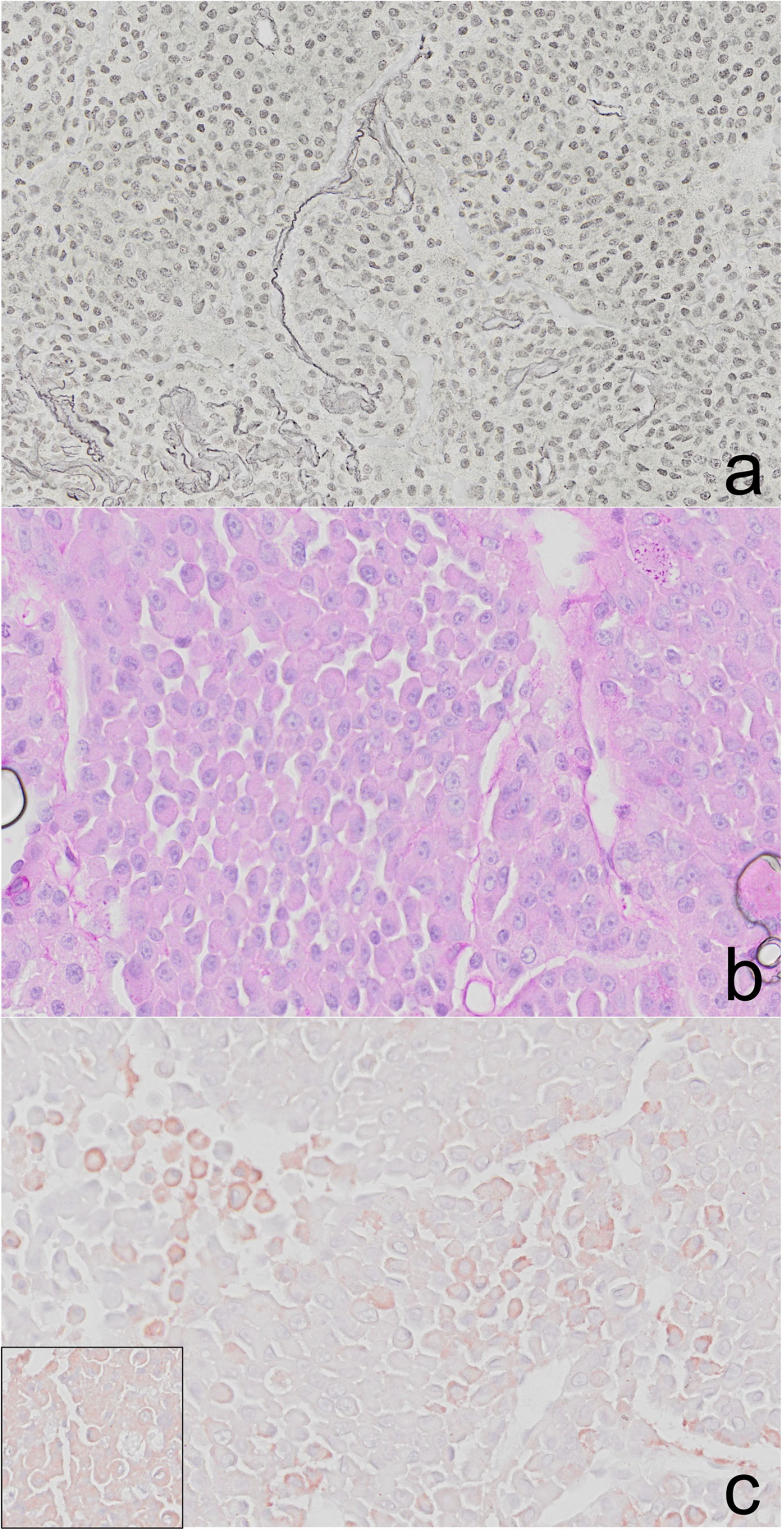
Pituitary adenoma, cat. **(a)** The reticulin network is lost within the neoplasm and is still present in the compressed, peripheral pituitary tissue. **(b)** The cytoplasmic granules within the adenoma lack staining with PAS. **(c)** Clusters of cells throughout the adenoma have faint, cytoplasmic immunoreactivity to growth hormone (GH). GH positive control cells (inset).

Histochemical staining with reticulin and periodic acid Schiff (PAS) and immunohistochemical staining with growth hormone were performed. Reticulin staining revealed a loss of reticular network within the neoplasm with respect to the compressed peripheral, non-neoplastic tissue (Figure 4), and the granules did not stain with PAS (Figure 4). Growth hormone immunohistochemistry revealed many clusters of cells with faint or weak cytoplasmic immunoreactivity within the neoplasm (Figure 4) similar to that in the control. The cellular architecture based on H&E staining, histochemical and immunohistochemical profile and lack of evidence of invasion or metastasis on histology and clinical imaging resulted in a diagnosis of somatotroph adenoma (9).

A week later, during post-surgical hospitalization, the patient's clinical condition worsened, and it died. A post-mortem examination with histopathology revealed fibrinosuppurative meningitis as a post-surgical complication, which was likely responsible for this patient's death. Other findings included a small focal area of thyroid nodular hyperplasia, vasculitis with thrombi in the lungs, multivacuolar degeneration, associated with focal amyloidosis and atrophy of the endocrine pancreatic islets, focally extensive lymphocytic enteritis, an incidental focal dilation in the esophagus and moderate, and diffuse vacuolar degeneration of the liver. Evaluation of remnant pituitary tissue in the sella turcica, as well as regional lymph nodes, confirmed a lack of invasion into the surrounding vasculature, parenchyma, bone, or lymphatics.

## Discussion

This is, to the best of the author's knowledge, the first cytologic description of a pituitary adenoma in animals. The cytologic appearance of the tumor exhibited some malignancy criteria such as marked anisokaryosis, prominent nucleoli, and nuclear molding. Despite these features, histological evaluation, in conjunction with CT and postmortem findings, was consistent with the accepted definition of an adenoma (9, 12). Cellular features are not a reliable indicator of malignant behavior in these neoplasms (12). The immunoreactivity to growth hormone and lack of PAS-positive granules supported a somatotrophic origin. Growth hormone immunoreactivity can vary widely in somatotroph adenomas, but the pattern and location of staining similar to that in the control (i.e., relatively weak in both the sample and the control) warranted the interpretation of immunoreactivity. The lack of intact reticulin network was used to differentiate adenoma from hyperplasia, and there was no evidence of vascular, bone, or local invasion or metastasis based on CT, gross postmortem examination, or postmortem histopathology (9). It is unclear why cytological interpretation in the smear showed cellular features of malignancy while histologically anisocytosis and anisokaryosis were interpreted as mild. This might implicate the higher specificity of Romanowsky-stained FNA samples to assess cytologic features, compared to histopathology. Furthermore, the lack of reference for malignant features in somatotrophs on cytology preparations or variation in cytologic features spatially within the tumor may have contributed to this discrepancy. While it is possible that this neoplasm is a carcinoma that has not yet metastasized, the prolonged clinical course of this patient's hypersomatotropism combined with the notion that carcinomas are less likely to be hormonally functional provide more evidence of a benign nature in this case (12).

Cytology of central nervous system tissues by direct aspiration, cannula, and squash preparation represents an option widely used in human medicine for the diagnosis of neoplasms such as medulloblastomas, germ cell tumors, and choroid plexus papillomas (13). It also seems to be a promising diagnostic technique in the veterinary neurosurgery field, given the recent advances in imaging diagnosis in that area (14). Accurate diagnosis *via* cytology was achieved in over 90% of human, canine, and feline cases in studies investigating over 4,000 correlated biopsies (15, 16).

Due to the complex range of biologic behavior patterns associated with pituitary tumors in people, the International Pituitary Pathology Club proposed pituitary adenomas, atypical adenomas, and carcinomas, in accordance with the developments involving the terminology of other neuroendocrine neoplasms, should be collectively referred to pituitary neuroendocrine tumors (17). In human pathology, the most updated classification of these tumors is mainly based on the tumor cell lineage, cell type, and related characteristics. It also involves the routine use of immunohistochemistry for pituitary transcription factors (PIT1, TPIT, SF1, GATA3, and ERα), but malignancy has always been associated with histologic evidence of regional or distant dissemination (18).

This patient had evidence of primary diabetes mellitus including islet cell pancreatic amyloidosis, however, diabetes mellitus in cats is considered a common consequence of hypersomatotropism, with hypophysectomy representing the gold standard type of therapy (19, 20). In a study, 59 out of 184 variably controlled diabetic cats had markedly increased (i.e., >1,000 ng/ml, RI = 208–443) serum levels of insulin-like growth factor 1 (21). The mechanism thought to play a role in this association is a GH-induced resistance to insulin at the level of adipocytes and myocytes (22).

Even though this patient was diagnosed with the non-thyroidal disease, which could account for the low total and free T4 serum levels, and the histopathologic evaluation of the thyroid gland revealed a small area affected by nodular hyperplasia, the TSH result was below the assay detection limit and this patient had evidence of pituitary atrophy secondary to the adjacent growth disorder. Also, thyroid nodular hyperplasia, even though highly prevalent, is reported to be non-functional in many cats (23). As such, a diagnosis of central hypothyroidism, due to pituitary atrophy and TSH deficiency, could not be ruled out. In fact, most human patients with active acromegaly have diminished serum TSH concentrations and around 25% have hypothyroidism (24, 25). All seven adult cats with primary spontaneous hypothyroidism in a recent case series had increased levels of TSH (26). Pituitary adenoma is listed as an important cause of central hypothyroidism in people (27), and the formal approach to human patients with clinical signs of hypothyroidism and concomitant pituitary disorders involves the investigation for central hypothyroidism (28).

Even though this was not further explored, the patient herein reported presented with polyuria. That could be merely attributed to osmotic diuresis secondary to glucosuria, but a defect with vasopressin (antidiuretic hormone, ADH) secretion by another pituitary gland impaired function as an additional contributor to the polyuria could be speculated.

One potential limitation of this report might be represented by the lack of cerebrospinal fluid (CSF) analysis done prior to surgery. As stated earlier, according to a recent WHO consensus, cerebrospinal involvement is used in the assessment of pituitary gland malignancy (29). However, this evaluation is still currently restricted to the histopathologic level, and not by using other tools, such as CSF cytologic analysis. Furthermore, the most recent WHO consensus only uses evidence of metastasis to differentiate the diagnosis of pituitary neuroendocrine tumors (18).

## Conclusion

Pituitary adenomas in cats may have a relatively aggressive cytologic picture and care should be taken not to presume malignancy. If tumor growth is causing secondary pituitary atrophy, resulting central endocrinopathies, such as hypothyroidism, should be ruled out.

## Data Availability Statement

The original contributions presented in the study are included in the article/supplementary material, further inquiries can be directed to the corresponding author/s.

## Author Contributions

FA and KN contributed to conception and design of the case description. FA organized the case data and wrote the first draft of the manuscript. KN and MC wrote sections of the manuscript. All authors contributed to manuscript revision, read, and approved the submitted version.

## Conflict of Interest

The authors declare that the research was conducted in the absence of any commercial or financial relationships that could be construed as a potential conflict of interest.

## Publisher's Note

All claims expressed in this article are solely those of the authors and do not necessarily represent those of their affiliated organizations, or those of the publisher, the editors and the reviewers. Any product that may be evaluated in this article, or claim that may be made by its manufacturer, is not guaranteed or endorsed by the publisher.
